# School Achievement and Oral Health Behaviour Among Adolescents in Finland: A National Survey

**DOI:** 10.3290/j.ohpd.a43349

**Published:** 2020-02-12

**Authors:** Anna-Emilia Lehtinen, Katja Joronen, Toni Similä, Anja Rantanen, Jorma I Virtanen

**Affiliations:** a Dentist, Oral and Dental Services, Health and Social Services, City of Pori, Pori, Finland. Drafted and wrote the manuscript.; b Adjunct Professor, Faculty of Social Sciences (Health Sciences), University of Tampere, Tampere, Finland. Wrote the manuscript.; c Statistician, Research Unit of Oral Health Sciences, Faculty of Medicine, University of Oulu, Oulu, Finland; Medical Research Center Oulu, Oulu University Hospital, Oulu, Finland. Performed the statistical analyses and wrote the manuscript.; d Adjunct Professor; Faculty of Social Sciences (Health Sciences), University of Tampere, Tampere, Finland. Wrote the manuscript.; e Adjunct Professor; Medical Research Center Oulu, Oulu University Hospital, Oulu, Finland; Department of Community Dentistry, University of Turku, Turku, Finland. Designed the study and wrote the manuscript.

**Keywords:** adolescents, health promotion, oral health behaviour, school achievement, toothbrushing

## Abstract

**Purpose:**

We examined oral health behaviour and its association with school achievement among Finnish adolescents.

**Materials and Methods:**

This study is part of the Finnish national School Health Promotion study (SHP). The study population comprised a representative sample of Finnish 15-year-olds (N = 45,877). A questionnaire inquired about the respondents’ school achievements and health habits (toothbrushing, smoking), background factors (age, gender, school type, family structure), and their parents’ background factors (education, smoking). Chi-square tests and logistic regression models were used in the statistical analyses.

**Results:**

Better school achievements were associated with better oral health behaviour: 73.1% of students with the highest mean grades (9–10) brushed their teeth twice daily, compared to 33.8% of those with the lowest mean grade (6.9 or less). The lowest mean grade was associated with brushing less than twice daily, especially among boys (odds ratios (OR) = 4.1; 95% CI 3.6–4.7) when compared to those with the highest mean grade, but also among girls (OR = 2.3; 95% CI 2.1–2.7). Smoking among boys was associated with poor oral hygiene (OR = 1.3; 95% CI 1.2–1.4).

**Conclusion:**

School success is strongly associated with oral health behaviour among adolescents. Preventive treatment should be targeted especially at boys with poor school achievement and smoking behaviour.

Oral health is a fundamental component of general health.^8^ Changes in health behaviour may be the result of, for example, growing up (development, skill development), learning, conditioning, or extrinsic and intrinsic rewards. The process is affected by many internal factors, such as knowledge, attitudes, intentions, and stress and external factors, such as social support and the environment.^42^ Both the school and the socioeconomic status of the family inﬂuence adolescent health behaviours.^33,43^

Toothbrushing is the most effective method of oral hygiene and the universally recommended frequency of brushing is twice a day.^29,30^ There are many clinical and psychological aspects that support this recommendation. Fluoride supplied via toothpastes and the removal of dental plaque are essential,^4^ but toothbrushing is also socially important, making teeth more attractive and removing mouth odours, and it seems to be the most important factor in influencing oral hygiene behaviour.

Already at the early stages of their lives, individuals begin to follow behavioural and educational tracks, leading to different positions in relation to health and social class.^23^ Young people who take care of their teeth behave in ways promoting other dimensions of health as well.^21^ Gender differences in brushing among adolescents have often been reported, with girls brushing more frequently than boys.^13,30^ Some studies’ authors have explained these gender behaviour differences according to the social and psychological impacts of oral health, ﬁnding that women perceive oral health as having a greater impact on their quality of life in general than men do.^30^ Whereas public awareness can aid in reducing and eventually eliminating oral health disparities, underserved and high-risk communities often do not perceive the necessity of oral health or the benefits conferred by regular dental care.^8^

Schools provide an effective platform for promoting oral health because they reach a substantial part of child population in the world. Several oral health problems are preventable, and their early onset is reversible.^17^ The World Health Organization (WHO) suggests that schools use a comprehensive programme – such as the ‘Oral Health Promoting School’ approach, where children can be provided with the school health policy, healthy school environment and oral health education (including smoking and oral hygiene) that enable them to make healthy decisions, adopt a healthy lifestyle and deal with conflicts.^17^ Commitment from central and local government, schools, families and the community is critical.

A recent review reported statistically significant inverse relationships between health-risk behaviours – such as tobacco use, inadequate physical activity and unhealthy dietary behaviours – and academic achievement among school-aged populations in the United States.^3^ The review indicated that the relationship between health-risk behaviours and low academic performance is ‘mutually reinforcing’. Ickovics et al suggest that schools may improve academic achievement by utilising non-traditional instructional strategies to improve student health.^15^

There is evidence of family factors – such as lower socioeconomic status (SES) in the family,^38,39^ lower education level in the family,^11^ and lower parental involvement^37^ – being associated with lower school achievement by pupils. However, the associations are both direct and indirect.^39^ Barr has suggested that both student and parent health should be considered in understanding SES-related disparities in academic achievement.^2^

Twice-a-day toothbrushing is a good indicator of a healthy lifestyle in general.^14^ It is plausible that parents of adolescents from high-income families are better educated than those from low-income families, and hence their knowledge of the importance of toothbrushing thereby exerts an influence on their children’s habit of brushing twice-daily from childhood.^36^ However, oral health habits also reflect individual factors independent of socioeconomic background.^21^ Social background seems to relate to the parents’ ability to promote their children’s education.

Little is known about the association between school achievement and oral health behaviour among youth. Thus, our aim in this study was to examine the prevalence of toothbrushing in Finnish adolescent boys and girls, and to study the associations between school achievement and oral health behaviour in boys and girls while controlling for SES. We used a large, nationally representative sample (N = 45,877) to provide an interesting contribution to the literature.

## Materials and Methods

### Subjects

This study uses data from the nationwide Finnish School Health Promotion study (SHP), which monitors the health, health behaviour, well-being and schooling of 14–20-year-olds. The SHP study is carried out nationwide every second year. Respondents include pupils in their eighth and ninth year of comprehensive school in mainland Finland and the Åland Islands, and the material covers 80% of these target groups in Finland.^31^ The survey is sent to every municipality in Finland, and each municipality decides if the schools in their area will participate in the survey.^18^ The Ethics Committee of the National Institute for Health and Welfare, Finland, approved the study. Participation in the study was completely voluntary and by answering, the student consented to the study.

The data was gathered by an anonymous and voluntary classroom-administered questionnaire under the supervision of a teacher in April 2013.^31^ In Finland, comprehensive school covers 9 years of education: school starts at the age of 7 and the basic education ends at the age of 16 years.

For the present cross-sectional study, we obtained data from 45,877 15-year-olds (not counting 830 participants who did not report their toothbrushing frequency) who were in the eighth (37%) or ninth (63%) grade of comprehensive school in the spring of 2013. Males comprised 49.9% of our study sample.

### The Questionnaire

The present study included only those participants who had reported their toothbrushing habits in the questionnaire. The question ‘How often do you brush your teeth?’ inquired toothbrushing frequency with the following answer options: never; less than once a week; at least once a week but not daily; once a day; or more than once a day. For our analyses, we formed three class (less than once a day, once a day, more than once a day) and dichotomised (less than twice a day, at least twice a day) variables according to the international recommendation of twice daily toothbrushing.^29^

The questionnaire inquired the participants’ gender and school grade. Students were asked to state their last term’s (December 2012) average grade score. The question ‘What was your average grade (for all subjects) on your latest school report?’ measured the adolescents’ school success with eight response options (less than 6.5, 6.5–6.9, 7.0–7.4, …, 9.5–10), which we narrowed to four categories (less than 7.0 (poor), 7.0–7.9 (satisfactory), 8.0–8.9 (good), 9.0–10 (excellent)).^35^ In Finland, the students are used to report their average scores and it is obvious that they remember them well. The question ‘Which of the following alternatives best describes your current smoking habits?’ assessed the adolescents’ smoking habits (among those who had ever smoked) with the response alternatives: I smoke once a day or more often; I smoke once a week or more often but not every day; I smoke less often than once a week; or I have quit smoking (temporarily or permanently). Respondents who answered the question ‘How many cigarettes, pipefuls of tobacco and cigars have you smoked altogether?’ with the answer option ‘none’ were identified as non-smokers. Based on these questions, we formed three categories for current smoking habits (current smoker, currently not a smoker, not at all). We further dichotomised this for the regression analyses (daily or occasional smoker, non-smoker).

The questionnaire also collected information on the participants’ parents. The question ‘During your life, have your parents smoked?’ assessed parents’ smoking habits (separately for the mother and father) with the answer options: never smoked; used to but has now quit; currently smokes; or I don’t know. We combined the smoking habits of the mother and father into a joint variable that considered the parents’ most prominent smoking habit with three categories (current smoker, currently not a smoker, not at all). We further dichotomised this for regression analyses (current smoker, non-smoker). The question ‘What is the highest educational level your parents have achieved?’ inquired the parents’ highest education (separately for the mother and father) with the following alternatives: primary or comprehensive school; upper secondary school or vocational education; occupational studies in addition to upper secondary school or vocational education; university, university of applied sciences, or other higher education institution; or no education. We further narrowed this information into three categories by combining the first and last options (basic education or less), as well as the second and third options (upper secondary school or vocational education with or without occupational studies). The questionnaire assessed the participants’ family structure with the question ‘Who are the adults you live with?’ and the following answer options: my mother and my father; my mother and my father alternately, my parents don’t live together; only my mother; only my father; my father/mother and his/her partner; one or more other adults; or none of the above. We dichotomised these alternatives into with both parents (mother and father) or other, assuming that the state of living with both parents would stand out from other structures in terms of health behaviour.^1^

### SHP Study

The SHP study collects information on Finnish adolescents’ health, health behaviour and related factors every second year.^31^ In contrast to earlier surveys, SHP covered participants from the whole country in 2013 (the participation rate was 84% among the adolescents in the eighth and ninth grades of comprehensive school). An anonymous, confidential, and voluntary classroom-administered questionnaire serves as the means of data gathering; the topics include living conditions, school conditions, health, health-related behaviour and school health services.

### Statistical Analysis

We first analysed the associations between background variables and toothbrushing frequency with cross tabulation, which we also did separately for boys and girls (due to a statistically significant difference in toothbrushing frequency among girls and boys). The chi-square test measured the statistical significance of the association between the variables. Multiple binary logistic regression served as the primary means of statistical analysis. We stratified the regression analyses by gender and presented the results with adjusted odds ratios (OR) and their 95% confidence intervals (95% CI) for mean grade, as the explanatory variable and confounders, which included parents’ (highest) education (according to the highest reported education for the mother/father), parents’ (most prominent) smoking habit, family structure and adolescent’s smoking status. The reference category of the dichotomised outcome was ‘brushing teeth at least twice daily’. We used IBM SPSS Statistics 22 for all statistical analyses.

## Results

Table 1 shows toothbrushing among 15-year-old Finns by their background factors. About half of the students (53.4%) followed the international recommendation of twice a day toothbrushing. A clear gender difference was observed: more girls (66.7%) brushed their teeth twice daily compared to the boys (40.1%). Parents’ higher education associated positively with toothbrushing among the 15-year-olds. In addition, living with both parents was also associated with better oral hygiene. Higher school achievement was associated with better toothbrushing behaviour: 73.1% of those students with the highest mean grades (9–10) brushed their teeth twice a day, while the percentage for those with a mean grade below 6.9 was 33.8%.

**Table 1:** Toothbrushing among Finnish 15-year-olds (%, n) by their characteristics^a^

	Toothbrushing			
	< once a day row % (n)	once a day row % (n)	> once a day row % (n)	Total column % (n)
**Gender**				
Boy	14.2 (3244)	45.7 (10,470)	40.1 (9177)	49.9 (22,891)
Girl	3.8 (882)	29.4 (6763)	66.7 (15,341)	50.1 (22,986)
**School grade**				
8th grade	9.8 (1659)	38.3 (6490)	51.9 (8808)	37.0 (16,957)
9th grade	8.5 (2467)	37.1 (10,743)	54.3 (15,710)	63.0 (28,920)
**Parents’ education**				
BEb or less	17.2 (459)	39.0 (1039)	43.8 (1166)	6.2 (2664)
SEc	9.2 (1895)	40.6 (8349)	50.2 (10,310)	48.1 (20,554)
TEd	7.0 (1368)	33.9 (6598)	59.1 (11,509)	45.6 (19,475)
**Family structure**				
Both parents	7.4 (2274)	36.9 (11,291)	55.6 (17,018)	68.0 (30,583)
Other	11.7 (1692)	39.1 (5630)	49.2 (7092)	32.0 (14,414)
**Mean grade**				
<6.9	19.2 (1281)	47.0 (3129)	33.8 (2254)	14.7 (6664)
7.0–7.9	10.9 (1695)	43.0 (6677)	46.0 (7143)	34.1 (15,515)
8.0–8.9	5.3 (877)	34.2 (5701)	60.5 (10,092)	36.7 (16,670)
9.0–10	3.3 (221)	23.6 (1565)	73.1 (4842)	14.6 (6628)

^a^ p <0.001 for all associations, chi-square test; ^b^ BE, basic education; ^c^ SE, high school, vocational school, occupational studies; ^d^ TE, university, polytechnics.

Table 2 presents 15-year-old Finnish boys and girls by their toothbrushing habits. School success was clearly related to boys’ toothbrushing frequency: 62.6% of those students with the highest mean grades brushed their teeth twice a day, while only 24.4% of those students with the lowest mean grade brushed twice daily (p <0.001). Altogether 18.4% of the boys with some experience of smoking brushed their teeth less than once a day. In addition, far fewer current-smoker boys brushed their teeth twice a day (30.2%) compared to non-smoking boys (44.7%). The most notable variables in the girls’ poor toothbrushing habits were poor academic performance and the mother’s low level of education (p <0.001). The majority (74.2%) of girls whose father had a university or polytechnic education brushed their teeth twice a day.

**Table 2:** Toothbrushing (%, n) among Finnish 15-year-old boys and girls by their characteristics^a^

	Boys				Girls			
	Toothbrushing				Toothbrushing			
	< once a day row % (n)	once a day row % (n)	> once a day row % (n)	Total column % (n)	< once a day row % (n)	once a day row % (n)	> once a day row % (n)	Total column % (n)
**Mean grade**								
<6.9	24.6 (1113)	51.0 (2309)	24.4 (1103)	20.0 (4525)	7.9 (168)	38.3 (820)	53.8 (1151)	9.4 (2139)
7.0–7.9	15.0 (1343)	49.3 (4430)	35.7 (3205)	39.6 (8978)	5.4 (352)	34.4 (2247)	60.2 (3938)	28.6 (6537)
8.0–8.9	8.3 (602)	42.2 (3061)	49.5 (3593)	32.0 (7256)	2.9 (275)	28.0 (2640)	69.0 (6499)	41.2 (9414)
9.0–10	7.5 (141)	29.9 (566)	62.6 (1183)	8.3 (1890)	1.7 (80)	21.1 (999)	77.2 (3659)	20.8 (4738)
Total	14.1 (3199)	45.8 (10,366)	40.1 (9084)	100 (22,649)	3.8 (875)	29.4 (6706)	66.8 (15,247)	100 (22,828)
**Father’s education**								
BEb or less	21.7 (574)	46.1 (1219)	32.2 (850)	12.9 (2643)	5.8 (164)	33.8 (958)	60.5 (1715)	13.6 (2837)
SEc	13.9 (1519)	48.4 (5301)	37.7 (4135)	53.6 (10,955)	3.9 (453)	31.4 (3621)	64.7 (7464)	55.2 (11,538)
TEd	10.1 (688)	41.4 (2831)	48.5 (3316)	33.5 (6835)	2.3 (152)	23.5 (1535)	74.2 (4847)	31.2 (6534)
Total	13.6 (2781)	45.8 (9351)	40.6 (8301)	100 (20,433)	3.7 (769)	29.2 (6114)	67.1 (14,026)	100 (20,909)
**Mother’s education**								
BEb or less	24.5 (455)	45.2 (840)	30.4 (565)	9.0 (1860)	6.4 (127)	35.7 (714)	57.9 (1157)	9.4 (1998)
SEc	13.8 (1511)	48.5 (5296)	37.7 (4120)	53.0 (10,927)	4.0 (452)	31.4 (3559)	64.6 (7320)	53.4 (11,331)
TEd	10.6 (831)	42.5 (3328)	46.9 (3675)	38.0 (7834)	2.6 (209)	24.4 (1924)	73.0 (5754)	37.2 (7887)
Total	13.6 (2797)	45.9 (9464)	40.5 (8360)	100 (20,621)	3.7 (788)	29.2 (6197)	67.1 (14,231)	100 (21,216)
**Family structure**								
With both parents	11.8 (1815)	45.6 (7001)	42.5 (6529)	68.7 (15,345)	3.0 (459)	28.2 (4290)	68.8 (10,489)	67.2 (15,238)
Other	18.5 (1290)	46.5 (3251)	35.0 (2449)	31.3 (6990)	5.4 (402)	32.0 (2379)	62.5 (4643)	32.8 (7424)
Total	13.9 (3105)	45.9 (10,252)	40.2 (8978)	100 (22,335)	3.8 (861)	29.4 (6669)	66.8 (15,132)	100 (22,662)
**Smoking**								
Current smoker	21.3 (1260)	48.5 (2876)	30.2 (1789)	26.3 (5925)	5.5 (318)	32.9 (1918)	61.7 (3598)	25.6 (5834)
Not currently	13.5 (468)	47.5 (1643)	39.0 (1351)	15.4 (3462)	3.7 (106)	29.4 (846)	66.9 (1923)	12.6 (2875)
Not at all	11.0 (1446)	44.2 (5796)	44.7 (5862)	58.3 (13,104)	3.2 (443)	28.1 (3943)	68.8 (9660)	61.7 (14,046)
Total	14.1 (3174)	45.9 (10,315)	40.0 (9002)	100 (22,491)	3.8 (867)	29.5 (6707)	66.7 (15,181)	100 (22,755)
**Parents’ smoking**								
Current smoker	18.8 (1385)	49.0 (3618)	32.2 (2376)	33.4 (7379)	5.7 (440)	33.5 (2591)	60.8 (4701)	34.4 (7732)
Not currently	13.7 (742)	46.0 (2487)	40.3 (2179)	24.5 (5408)	3.6 (196)	29.1 (1605)	67.3 (3707)	24.5 (5508)
Not at all	10.3 (962)	43.6 (4053)	46.1 (4284)	42.1 (9299)	2.4 (220)	26.3 (2419)	71.4 (6573)	41.0 (9212)
Total	14.0 (3089)	46.0 (10,158)	40.0 (8839)	100 (22,086)	3.8 (856)	29.5 (6615)	66.7 (14,981)	100 (22,452)

^a^ p <0.001 for all associations, chi-square test; ^b^ BE, basic education; ^c^ SE, high school, vocational school, occupational studies; ^d^ TE, university, polytechnics.

Table 3 shows adjusted ORs for variables affecting toothbrushing habits for boys and girls. Poor school success was strongly associated with poor toothbrushing for both genders. A low mean grade (6.9 or less) was associated with brushing less than twice a day, especially among boys (OR = 4.1; 95% CI 3.6–4.7) when compared to those with the highest mean grade (9–10), but also among girls (OR = 2.3; 95% CI 2.1–2.7). Furthermore, a mean grade of 7.0–7.9 was significantly associated with poor oral hygiene for boys (OR = 2.7; 95% CI 2.4–3.0) and for girls (OR = 1.9; 95% CI 1.7–2.1).

**Table 3:** Logistic regression model for essential variables^a^ affecting 15-year-old boys’ (n = 19,772) and girls’ (n = 20,696) toothbrushing habits^b^

	Boys						Girls					
					95 % CI						95 % CI	
	B	SE	Sig.	OR	Lower	Upper	B	SE	Sig.	OR	Lower	Upper
**Mean grade**												
≤6.9	1.416	0.066	<0.001	4.120	3.618	4.693	0.851	0.065	<0.001	2.341	2.063	2.657
7.0–7.9	0.986	0.056	<0.001	2.681	2.401	2.995	0.650	0.047	<0.001	1.915	1.745	2.101
8.0–8.9	0.506	0.056	<0.001	1.658	1.487	1.850	0.323	0.043	<0.001	1.381	1.269	1.503
9.0–10 (ref.)			<0.001	1.0					<0.001	1.0		
**Parents’ education**												
BEc or less	0.358	0.070	<0.001	1.430	1.246	1.641	0.354	0.064	<0.001	1.425	1.259	1.614
SEd	0.178	0.032	<0.001	1.194	1.123	1.271	0.269	0.032	<0.001	1.309	1.228	1.394
TEe (ref.)			<0.001	1.0					<0.001	1.0		
**Family structure**												
With both parents (ref.)				1.0						1.0		
Other	0.059	0.034	0.082	1.061	0.992	1.134	0.104	0.033	0.002	1.110	1.040	1.184
**Smoking**												
Daily or occasional	0.234	0.037	<0.001	1.263	1.174	1.359	0.026	0.037	0.486	1.026	0.955	1.103
Non-smoker (ref.)				1.0						1.0		
**Parents’ smoking**												
Current	0.239	0.035	<0.001	1.271	1.187	1.360	0.194	0.033	<0.001	1.215	1.138	1.297
Non-smoker (ref.)				1.0						1.0		
Constant	–0.664	0.051	<0.001	0.515			–1.382	0.039	<0.001	0.251		

^a^ The variables with strongest associations were included in the model; ^b^ presented estimates are adjusted and the reference category for the outcome variable was ‘at least twice daily’; ^c^ BE, basic education; ^d^ SE, high school, vocational school, occupational studies; ^e^TE, university, polytechnics.

Parents’ current smoking was positively associated with poor brushing habits for both genders (OR = 1.3; 95% CI 1.2–1.4 for boys and OR = 1.2; 95% CI 1.1–1.3 for girls; Table 3). Smoking among boys was associated with poor oral hygiene (OR = 1.3; 95% CI 1.2–1.4 for daily or occasional smoking), but the relation was not statistically significant among girls.

Parents’ education level was associated with children’s toothbrushing habits (Table 3). Toothbrushing was poorest in children from families where the parents’ education level was basic education or less (OR = 1.4; 95% CI 1.2–1.6 for boys and OR = 1.4, 95% CI 1.3–1.6 for girls).

## Discussion

This study showed that school achievement seems to have a strong association with oral health behaviour among adolescents while controlling for family factors and smoking behaviour. Daily or occasional smoking was shown to be associated with a lower frequency of toothbrushing among boys only. Parents’ smoking was associated with lower toothbrushing among boys and girls; this association remained when controlling for other family factors and school achievement. Furthermore, the lower education level of parents seems to be connected to their children’s less frequent toothbrushing.

Our study shows that school success is significantly associated with toothbrushing activity among Finnish adolescents. Similar findings have been reported earlier in the Nordic countries.^13,21,24^ Toothbrushing frequency has been found to be associated with attained education level via school success and goal-directed behaviour.^21^ Honkala et al reported an association between poor school grades and health-damaging behaviour.^13^ In turn, health-enhancing behaviour, such as physical activity, has been shown to be associated with better academic achievement.^24^ Students who are assessed to be having problems at school (eg, anxiety or difficulties concentrating) also have various risk factors for future mental and physical health and social exclusion.^26^ By monitoring students’ school performance and grades, teachers and school staff can tentatively infer their students’ oral health behaviour.^17^

Studying a theoretical upper secondary programme has been found to be a statistically significant explanatory variable for investments in oral health.^9^ One reason for this could be that individuals studying such programmes are more accustomed to dealing with and embracing instructions and information. Moreover, academic self-efficacy has a strong relationship with academic achievement. Young people who believe in their capabilities to exercise control over their educational performance achieve higher results academically than counterparts who have less efficacious beliefs in their academic pursuits.^5^ A study in six European countries showed that high motivation for education and high self-esteem also correlate with good grades.^35^ We suggest that adolescents with excellent grades may take care of their duties in school and in life generally while those with poor grades need more targeted help with preventive methods against oral diseases.

Already in early adolescence, health behaviours distinguish those children whose educational career is likely to be less favorable.^22^ Adolescents who neglect their dental hygiene may endanger their social value and acceptance among their peers, and consequently this may indicate potential problems in the future.

In our study, a clear gender difference was observed: the girls tended to follow the recommended toothbrushing frequency more often than the boys did, which is in line with previous international studies.^14,29,30^ About two-thirds of the girls (67%) brushed their teeth twice daily, while the corresponding percentage for the boys was 40%. The association of gender difference and health behaviour is stronger when adjusting for differences in the families’ financial circumstances and parental occupational status.^30^ Girls commonly adopt the stable, more than once-a-day toothbrushing frequency much earlier than boys do.^25,27^ Our findings support this argument, however the gender difference varied according to socioeconomic group. Nevertheless, the girls always brushed as recommended more often when compared with boys who were achieving the same grades. A small improvement in brushing activity among Finnish adolescents could also be seen: in 2010–2011 a smaller proportion of adolescents brushed their teeth twice a day (girls 62%, boys 38%) compared with our findings.^41^ Perhaps the most notable finding in our study was that the boys with the lowest mean grades (6.9 or less) in particular have greater problems with proper oral hygiene: one-quarter of the boys brushed their teeth less than once a day. This group of boys is clearly in great need of targeted health promotion and preventive efforts.

This study also indicated that adolescent smoking was related to worse oral health habits among boys. Smoking has been shown to be associated with bad oral health behaviour, as well as with caries and periodontal diseases.^43,44^ Users of one particular tobacco product, such as cigarettes or snus (a snuff product popular in the Nordic countries), have a more positive attitude towards other tobacco products.^20,43^ An accumulation of smoking and snus use may also signal an accumulation of other lifestyle-risk factors, such as a drug-taking and risk-seeking lifestyle. Possible explanations for this could be, for example, depressive symptoms or other mental health problems.^7,43^

According to a previous study, smoking and toothbrushing frequencies seem to be related among both genders and are independent of age: adolescents between 12 and 18 years who reported brushing their teeth less than twice daily smoked more frequently than adolescents who brushed their teeth according to the recommendations.^13^ However, in our study, girls’ smoking and toothbrushing habits were not significantly associated. Girls in Finland value oral health more than boys and practice better oral hygiene regardless of their smoking status.^32^ On the other hand, poor school success is related to smoking habits: adolescents who smoke have lower school achievement and are more influenced by peers compared to adolescents with excellent school achievement.^35^ Earlier, boys have smoked more compared to girls,^6^ but the difference between the boys’ and girls’ smoking rate has narrowed during the last decade, and currently girls smoke more than boys in many Western countries,^35^ such as in Sweden.^12^ In addition, adolescents’ smoking has been shown to be associated with parents’ smoking,^35,43^ and this was the case in our study too.

Finnish adolescents have been among the most infrequent toothbrushers in Europe, although the situation has improved in recent years.^14,29^ According to the Health Behaviour in School-aged Children (HBSC) study (2001/2002), only 45% of Finnish 15-year-olds brushed their teeth twice a day, while 80% of Danish, 81% of Norwegian, and 83% of Swedish 15-year-olds brushed their teeth as recommended.^29^ In a later HBSC study (2005/2006), only half of the Finnish 15-year-olds (50%) brushed their teeth twice daily. Overall, oral hygiene habits among Finnish adolescents have moved towards the universally recommended twice daily practice, but they remain inferior when compared to the other Nordic coevals.^6,14^ Historically lower level of oral health and cultural aspects might explain these differences. Behaviours are adopted at a very young age, but at the age of 15 years, adolescents have the main responsibility for their own oral health.^32^

The recent Program for International Student Assessment (PISA) results indicate that Finns continue to score very highly in science.^34^ Finland also appears to be the only Organization for Economic Co-operation and Development (OECD) country where girls outscore boys. This might be a reason for better oral health behaviour among Finnish girls.

Unlike other parts of Europe, the Nordic countries have comprehensive public dental services, and between 80 and 95% of children are seen by a dentist or a dental hygienist regularly.^45^ It is still unclear why free dental care in Finland does not seem to have the expected impact on oral health habits among boys.

One of the main findings of this study with implications to health promotion activities is the association between lower school achievement and lower frequency of toothbrushing. Similar findings have been reported by Jackson et al among United States schoolchildren.^16^ In addition, studying a theoretical upper secondary programme may be a statistically significant variable for investments in oral health.^9^ The present study also indicated that adolescent smoking is related to worse toothbrushing habits only among boys. The result is partly congruent with the findings of a recent review that found smoking to be associated with lower academic achievement among school-aged children.^3^

Toothbrushing was worse in children from families where the parents’ education level is basic education or less. This might indicate that parents with higher education are more knowledgeable regarding the importance of toothbrushing.^36^ The educational level achieved seems to be a statistically significant predictor of a wide range of health outcomes, including health status in later life.^10^ Cognitive, behavioural and motivational factors are of major importance in health promotion.^28^ Additionally, several behavioural risk factors – such as smoking and unhealthy diets – appear to contribute to health inequalities (ie, risk behaviours have been more prevalent among lower socioeconomic groups). Adolescence may be a critical period for later health because risk behaviours, once initiated, often track into adulthood.^33^ Health education has the potential to help students maintain and improve their health, prevent disease and reduce health-related risk behaviours.^19^

The Finnish School Health Promotion study reaches approximately 80% of Finnish 15-year-olds.^43^ The gender distribution in the study was equal, and because of the large sample size in our study, the findings may be generalised to all Finnish 15-year-old adolescents. The large sample size also makes our study comparable to international surveys studying adolescents’ oral health behaviour. Self-reported outcome measures might be susceptible to socially desirable answering,^40^ but the students participated in this study voluntarily and the answers were anonymous, so the responses can be considered reliable.

## Conclusions

In conclusion, school success is strongly associated with oral health behaviour among adolescents. Schools ought to provide a supportive environment for promoting health of children. Oral health promotion should be targeted especially at boys with poor school achievement and smoking behaviour.
